# First dorsal metacarpal artery (FDMA) flaps: A novel classification system incorporating various modifications and subtypes—a 5 year retrospective comparative study

**DOI:** 10.1016/j.jpra.2025.08.019

**Published:** 2025-08-22

**Authors:** Mir Yasir, Hillal Ahmad Bhat, Eidan Bilal, Mir Mohsin, Peerzada Umar Farooq, Haroon Rashid Zargar, Altaf Rasool, Jaswinder Kaur, Mohsin Saleem Khan, Sheikh Adil Bashir, Adil Hafeez Wani

**Affiliations:** a Department of Plastic Surgery, Sher-i- Kashmir Institute of Medical Sciences, Srinagar, Kashmir 190011, India; b Department of Biochemistry, GMC Baramullah, Kashmir 193101, India

**Keywords:** First dorsal metacarpal artery (FDMA) flap, Thumb reconstruction, Extended FDMA flap, FDMA flap classification

## Abstract

**Introduction:**

The first dorsal metacarpal artery (FDMA) flap, including its extended versions, is a reliable and versatile option for reconstructing thumb defects. However, a structured and practical classification system for its various modifications has been lacking.

**Objectives:**

This study aimed to assess the versatility of the FDMA flap and its modifications in managing a range of thumb defects and to propose a novel, clinically applicable classification system to facilitate flap selection and improve outcomes.

**Methods:**

A retrospective observational study was conducted over five years at a tertiary care referral hospital with a specialized hand unit. A total of 29 patients with thumb defects underwent FDMA flap reconstruction. Flaps were classified into five types:

• Type I Islanded flap, up to PIP joint; subtypes It (tunneled) Is (split skin)
• Type II Peninsular flap, up to PIP joint
• Type III Extended islanded flap beyond PIP joint; subtypes IIIt (tunneled), IIIs (split skin),
• Type IV Extended peninsular flap beyond PIP joint, and
• Type V Other modifications, e.g., reverse flaps or bilobed flaps (not used in this study).
• Data on flap survival, complications, hospital stay, and 6-month patient satisfaction were analyzed.

**Results:**

Defect sizes ranged from 2 × 3.5 cm to 4 × 5.5 cm. The majority of patients were young male industrial and carpentry workers. Flap distribution was as follows: Type I (12 cases: 9 It, 3 Is), Type II (4 cases), Type III (7 cases: 4 IIIt, 3 IIIs), and Type IV (6 cases). Flap survival was highest in peninsular and split-skin flaps, while Type IIIt flaps showed the highest complication rate (odds ratio [OR] 5.0, *p* = 0.03). Overall, 79.3 % of patients reported high satisfaction at 6 months.

**Conclusion:**

The FDMA flap, including its various modifications, remains a versatile and reliable option for thumb reconstruction. The proposed classification system offers a simple, clinically relevant framework to guide flap selection based on defect characteristics, which may help minimize complications and improve clinical outcomes.

## Introduction

The human hand is an intricately designed and remarkably functional organ. Restoring this beautiful architecture and complicated design following an injury is critical for attaining optimal outcomes. Given the crippling disability associated, hand injuries, howsoever trivial, require urgent and specialized evaluation. Injury to the thumb has the maximum impact on the functioning of the hand.^1^

The routine activities of the hand, especially grasping and precision handling, require well-coordinated movements of the thumb with other digits.^2^ The paucity of local soft tissue, coupled with the requirement for a reliable, pliable, and sensate flap for soft tissue coverage of compound defects of the thumb, makes reconstruction extremely challenging for the surgeon.^3^

Hilgenfeldt’s sensate dorsal metacarpal artery flap (FDMA) for thumb defects was further modified by Holevich.^4^^,^^5^ Foucher and Braun described the first neurovascular island flap.^6^ Gebhart and Meissl extended the distal limit of the flap into the middle phalanx and described a wraparound extended FDMA flap for circumferential defects.^7^

The first dorsal metacarpal artery originates from the radial artery just before its bifurcation in the first intermetacarpal space. It passes over the fascia of the first dorsal interosseous muscle in most cases, while in some it may be subfascial. The artery further divides into three main branches: ulnar, intermediate, and radial. It is the ulnar branch that supplies the FDMA flap by ramifying distally and supplying the dorsal aspect of the index finger.

This study aimed to assess the versatility of the FDMA flap and its various modifications in managing a wide range of dorsal, volar, and oblique thumb defects. Additionally, we propose a novel, structured, and clinically relevant classification system to guide flap selection and improve surgical decision-making. We analyzed outcomes across flap types, focusing on survival, complications, hospital stay, and 6-month patient satisfaction.

Clinical Relevance: The proposed classification system offers a simple, structured, and easily remembered approach to FDMA flap selection. By categorizing flaps based on their application and extension, it provides a systematic decision-making tool for hand surgeons. This classification consolidates existing variations into a single framework, aiding young surgeons in understanding flap dynamics, survival potential, and associated complications with each subtype. The surgeon can thus choose an appropriate flap subtype after properly assessing the requirements of the defect, their technical comfort, and experience, to achieve successful results. Complications can often be avoided by selecting a safer flap even if extended designs are required to cover extensive (volar or dorsal) thumb defects.

## Materials and methods

This retrospective study was conducted over a 5-year period (September 2019 to August 2024) at a tertiary care referral hospital with a specialized hand unit managing diverse hand injuries. A total of 29 patients with thumb defects requiring flap coverage were included in the study.

Inclusion Criteria: Patients with thumb defects with exposure of critical structures (bone, joint, tendon) or pulp loss necessitating flap cover.

Exclusion Criteria: Patients with comorbidities affecting flap survival, such as:

• Peripheral vascular disease (PVD)
• Diabetes mellitus
• Chronic smoking history affecting microvascular circulation
• Patients with infected wounds at the time of surgery
• Patients with previous local flap procedures in the affected region

Flap Selection Criteria: All patients underwent surgery after obtaining informed consent. Flap selection was determined primarily by defect characteristics (size, location, distal extent), exposure of critical structures, and patient factors (age, occupation, comorbidities). Surgeon experience and technical comfort also played a role, especially for extended or tunneled flaps.

FDMA flaps were classified into five main types: (Yasir's classification of FDMA flaps)

**Figure 1:**
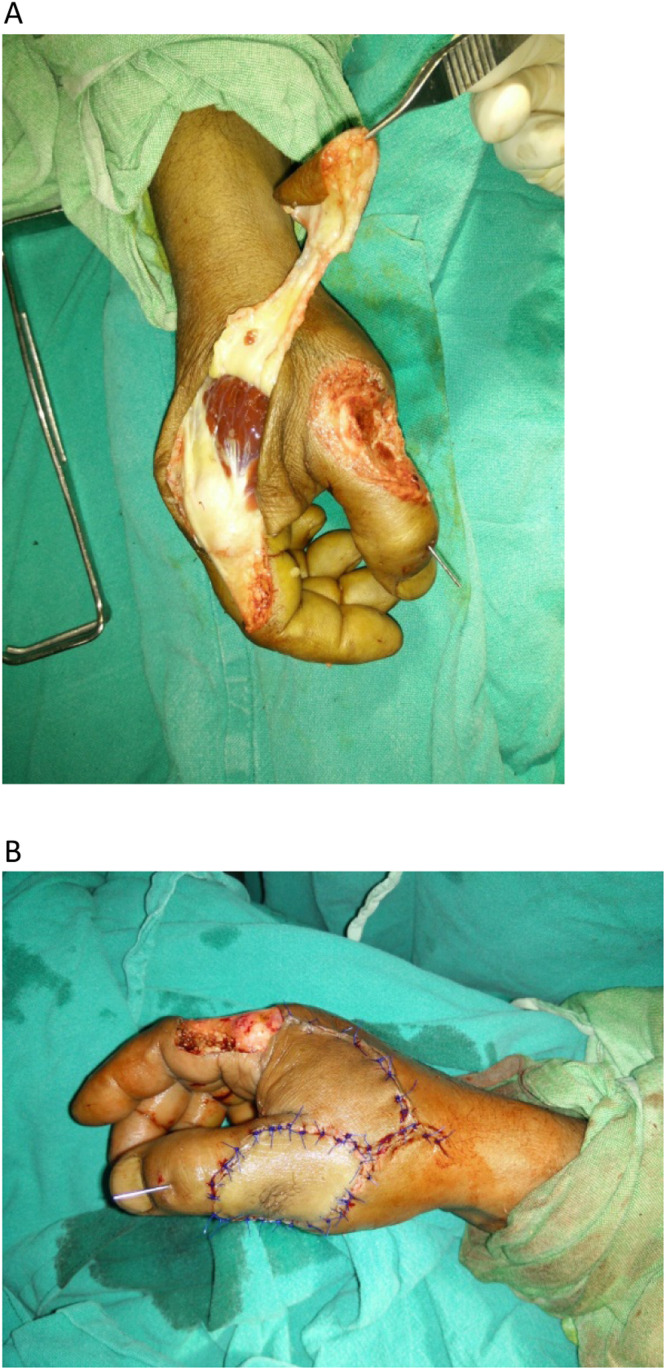
Conventional FDMA flap. A: Machine injury Thumb with exposed MCP joint, Conventional FDMA Flap harvested for cover. B: Flap inset into the defect.
• It: Flap tunneled into the defect
• Is: Intervening skin is split
• Type II: Conventional Peninsular FDMA Flap (skin pedicle, and distant extent up to PIP joint) (Figure 2)

• Type I: Conventional Islanded FDMA Flap (subcutaneous pedicle and distant extent up to PIP joint) (Figure 1)

**Figure 2:**
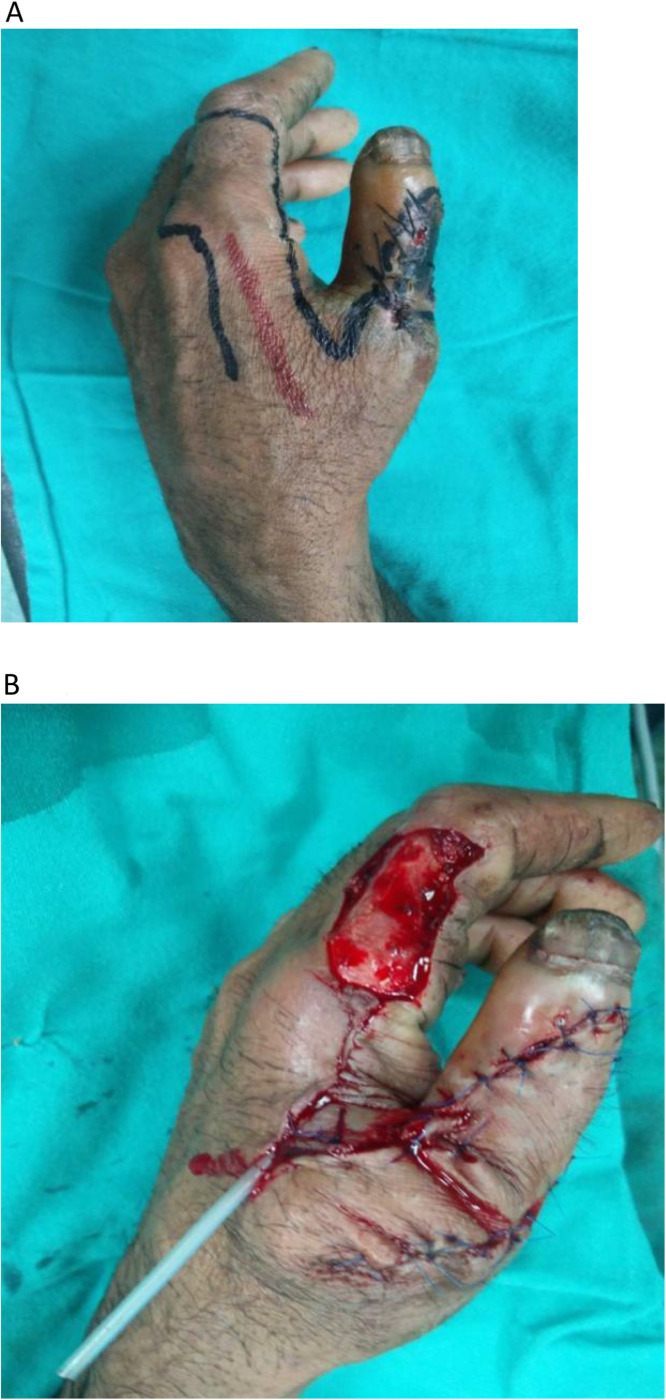
Peninsular FDMA flap. A: Crush Injury with necrosed skin, Planning done for Peninsular FDMA flap. B: Flap covering the defect.
• Type III: Extended Islanded FDMA Flap (subcutaneous pedicle, and distant extent beyond PIP joint) (Figure 3)

**Figure 3:**
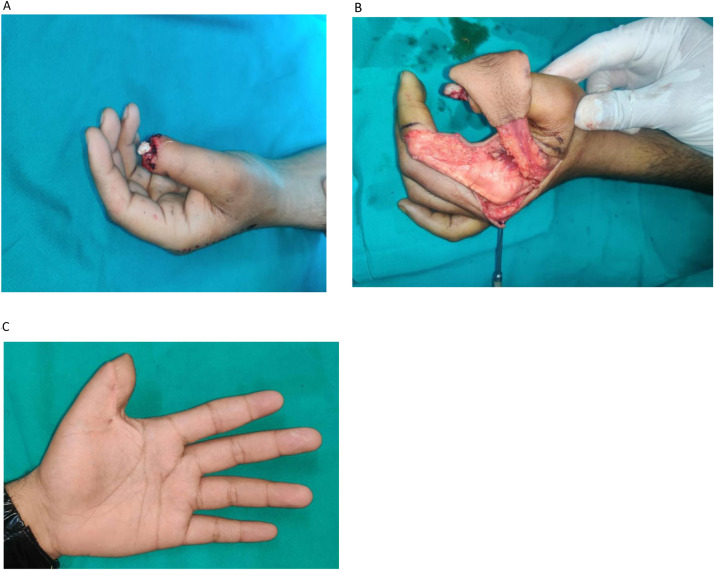
Extended FDMA flap. A: Post traumatic circumferentially exposed distal phalynx. B: Extended Flap harvested for coverage of defect. C: Follow picture of the patient showing well settled flap.
• IIIt: Flap tunneled into the defect
• IIIs: Intervening skin is split
• Type IV: Extended Peninsular FDMA Flap (skin pedicle, and distant extent beyond PIP joint) (Figure 4)

**Figure 4:**
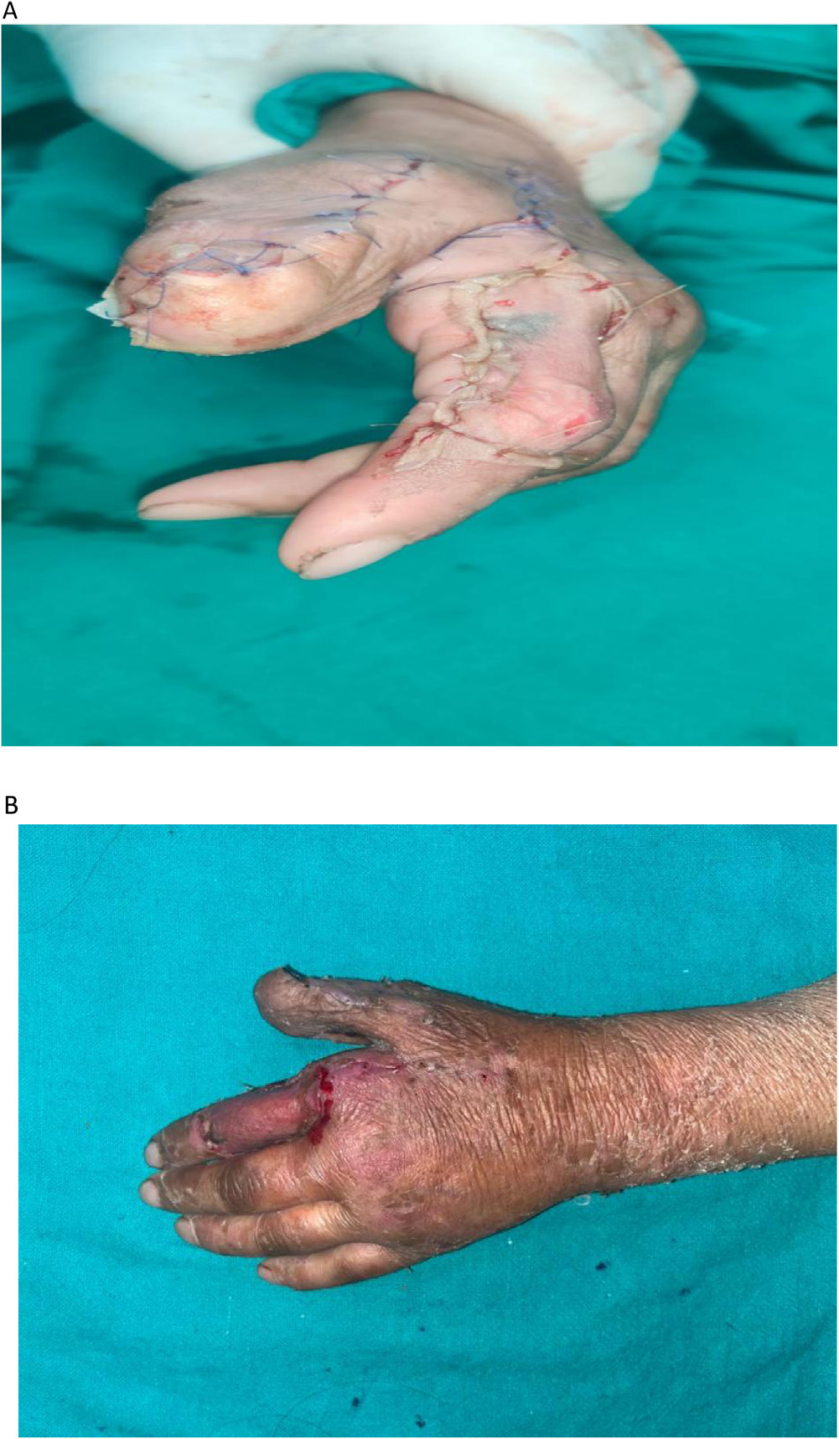
Extended peninsular FDMA flap. A: Extended FDMA inset into the defect. B: Immediate follow-up of the patient.
• Type V: Other modifications (reverse FDMA flapsor bilobed FDMA flaps)

**Figure 5A:**
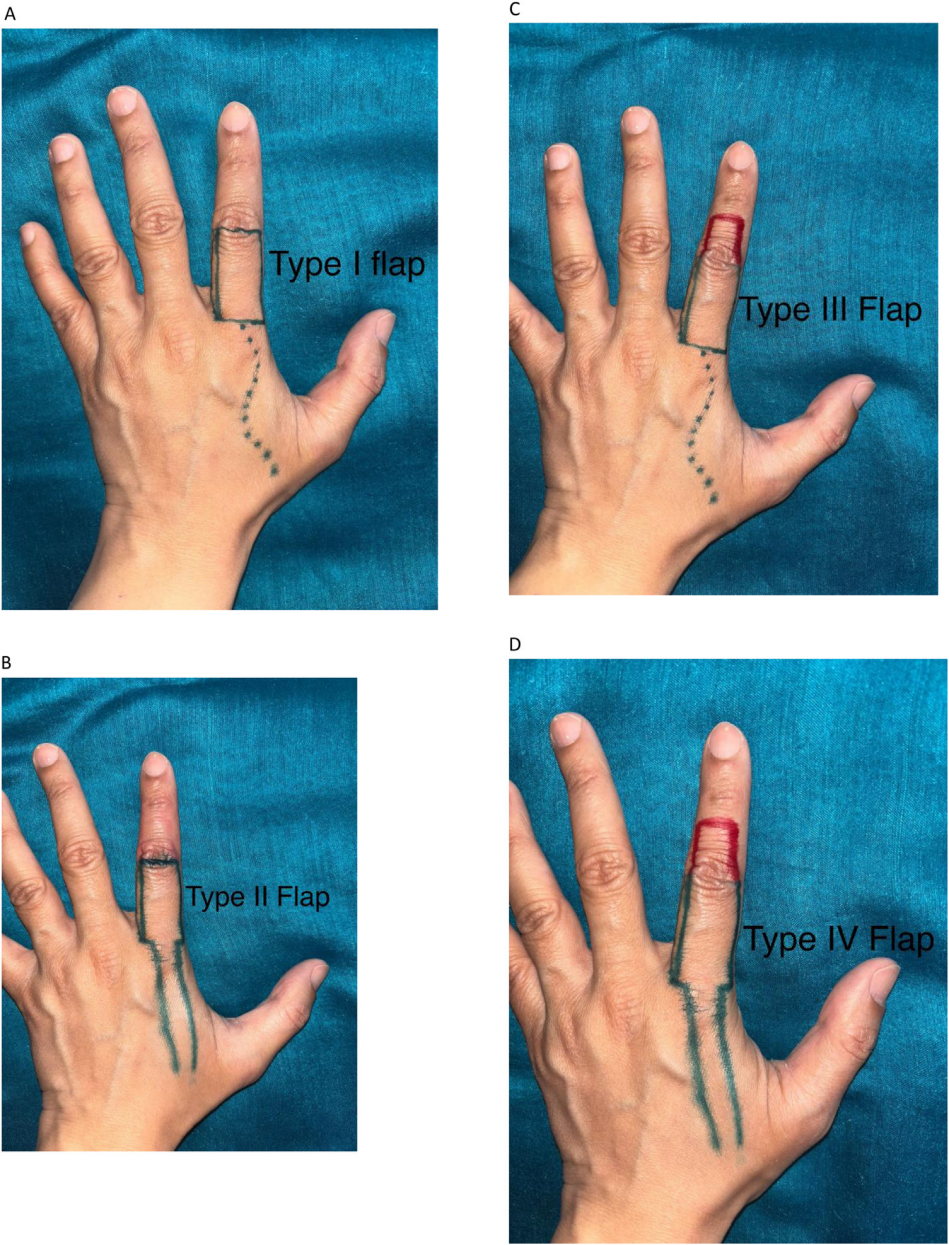
Type I flap illustrating classic islanded FDMA WITH PIP as distal extent. B: Type II flap, the peninsular variety and PIP as distal extent. C: Type III flap, extending beyond PIP (IN RED) and islanded D: Type IV flap extending beyond PIP and peninsular skin paddle.

For clarity of classification, we have included illustrative figures depicting the different flap types (Figure 5a–d)

Type I and Type II flaps were conventional flaps, in the sense that the proximal interphalangeal (PIP) joint formed the distal boundary of the flap, whereas Type III and Type IV were extended flaps whose distal extent crossed the PIP boundary into the middle phalanx.

Further, as the names suggest, Type I and Type III flaps were islanded flaps in which subdermal dissection was carried out, and the pedicle consisted of subcutaneous tissue and fascia over the first dorsal interosseous muscle. The flap was either tunneled (It and IIIt) into the defect or the intervening skin was split (Is and IIIs) to accommodate the pedicle. Type II and Type IV flaps were peninsular flaps in which the pedicle consisted of skin, subcutaneous tissue, and fascia over the muscle. Type V flaps included other modifications described in the literature, such as the reverse FDMA flap and bilobed FDMA flap.^8^^,^^9^ Type V flaps were not performed in this study.

Note: Subtypes It, Is, IIIt, and IIIs represent technical refinements within Types I and III, selected based on flap tension, ease of inset, defect geometry, and surgeon expertise. These technical decisions were found to influence flap survival and complication rates in our results.

Patient Satisfaction Assessment: Patient satisfaction was evaluated at 6 months postoperatively using a 5-point Likert scale: 1 = very dissatisfied, 2 = dissatisfied, 3 = neutral, 4 = satisfied, 5 = very satisfied. Scores of 4 or 5 were categorized as high satisfaction and analyzed in relation to flap survival and complications.

Statistical Analysis: The data were interpreted using SPSS v. 23.0. Logistic regression analysis was used to calculate probabilities. A *p*-value <0.05 was taken as significant (Table 1, 2, 3).

**Table 1:** Stratification of FDMA flap with respect to survival.

Classification	Total (N)	Survival		OR (95% CI)	P value
		Survived N (%)	Lost N (%)		
II	04	04 (100.0)	00 (0.0)	**Reference Group (OR=1.0)**	
It	09	08 (88.8)	01 (11.2)	1.1 (0.07–1.6)	0.7
Is	03	03 (100.0)	00 (50.0)	1.25 (0.06–2.6)	0.7
IIIt	04	02 (50.0)	02 (50.0)	5.0 (0.3–7.2)	0.5
IIIs	03	03 (100.0)	00 (0.0)	1.25 (0.05–1.6)	0.7
IV	06	05 (83.3)	01 (16.7)	1.6 (0.1–2.4)	0.6

**Table 2:** Stratification of FDMA flap with respect to complication.

Classification	Total	Complication		OR (95 % CI)	P value
		No N (%)	Yes N (%)		
II	04	04 (100.0)	00 (0.0)	**Reference Group (OR=1.0)**	
It	09	07 (77.7)	02 (22.3)	0.9 (0.2–2.3)	0.5
Is	03	03 (100.0)	00 (0.0)	1.25 (0.05–2.6)	0.7
IIIt	04	02 (50.0)	02 (50.0)	5.0 (0.3–7.7)	**0.03**^⁎^
IIIs	03	02 (50.0)	01 (50.0)	3.3 (0.2–5.5)	0.5
IV	06	05 (83.3)	01 (16.7)	1.6 (0.1–2.4)	0.6

* Statistically significant.

**Table 3:** Stratification of FDMA flap with respect to hospital stay.

Classification	Total	Hospital stay		OR (95% CI)	P value
		Short stay N (%)	Long stay N (%)		
II	04	04 (100.0)	00 (0.0)	**Reference group (OR=1.0)**	
It	09	07 (77.7)	02 (22.3)	1.8 (0.2–2.3)	0.5
Is	03	02 (66.6)	01 (33.4)	3.3 (0.2–5.4)	0.4
IIIt	04	02 (50.0)	02 (50.0)	5.0 (0.3–7.7)	0.5
IIIs	03	02 (66.6)	01 (33.4)	3.3 (0.2–5.4)	0.5
IV	06	04 (66.6)	02 (33.4)	3.0 (0.2–3.6)	0.6

## Results

The defects ranged from 2 × 3.5 cm to 4 × 5.5 cm. The majority of cases were young males working in machine industries and carpentry. One 71-year-old female had squamous cell carcinoma involving the dorsum of the proximal phalanx of the thumb. Of the 29 patients, 24 were male and 5 were female, with an age range of 16 to 71 years.

A total of 12 patients underwent Type I flap, of which 9 were tunneled (Type It) and 3 had intervening skin split (Type Is). In 7 patients, an Extended Islanded FDMA flap (Type III) was performed, where the flap was tunneled in 4 cases (Type IIIt) and intervening skin was split in 3 cases (Type IIIs). Four patients were managed using the conventional FDMA with skin bridge (Type II flap), while 6 patients underwent the Extended FDMA flap with skin bridge (Type IV).

On comparison of the results, it was found that out of 9 Type It flaps, 7 survived completely, while 2 flaps developed postoperative edema and congestion, of which one was lost completely, and another was salvaged by releasing a few sutures, elevation, and massage. There was no significant postoperative complication in Type Is cases, although one had a suture site infection.

All Type II flaps survived with no complication.

Among 4 Type IIIt flaps, two patients developed postoperative edema and congestion. The congestion worsened over subsequent days, and a portion of the flap was lost in both patients. Both were managed conservatively, but these patients required prolonged hospital stays and frequent outpatient visits for the management of the residual defect. Out of three Type IIIs patients, one developed postoperative edema, but all three flaps survived completely.

Out of six Type IV flaps, four survived completely; one patient developed a suture site infection, and one had partial flap loss following flap congestion.

**Figure 6:**
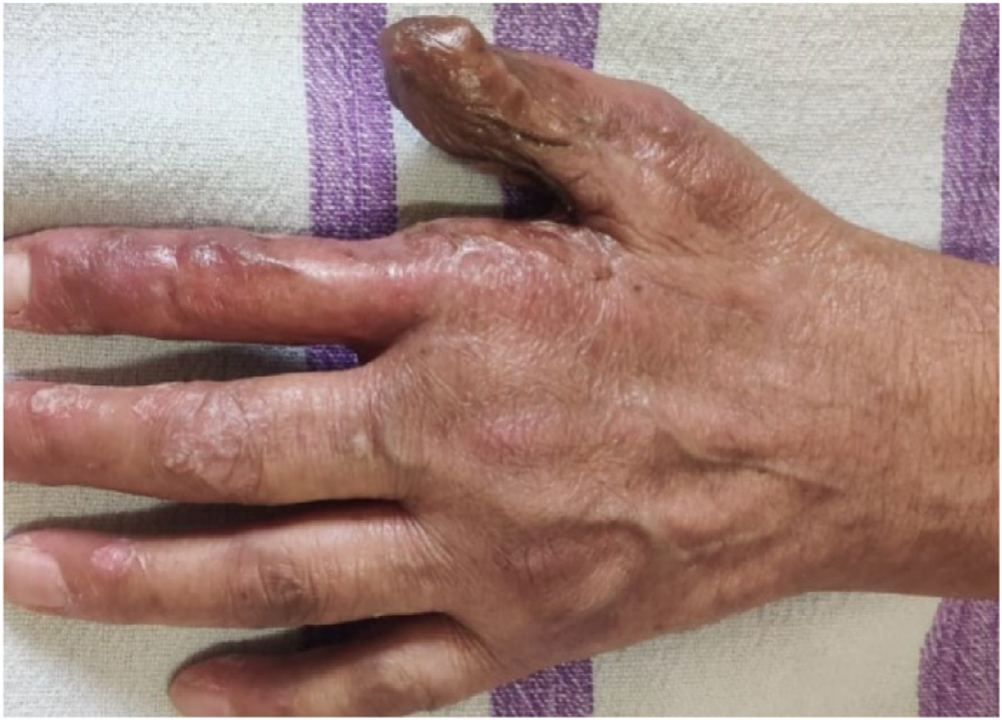
1 year follow-up of a patient with Extended FDMA showing well settled donor scar.

**Figure 7:**
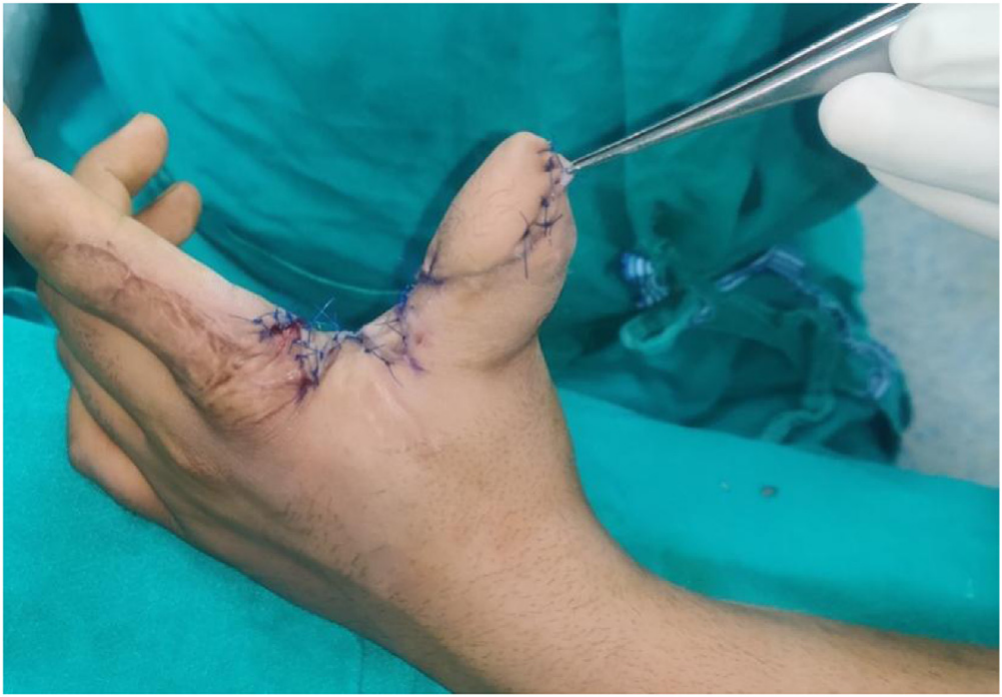
Secondary procedure (flap thinning and web space widening).

Patient satisfaction scores were evaluated at 6 months. A total of 23 out of 29 patients (79.3%) reported satisfaction scores of 4 (satisfied) or 5 (very satisfied) on the 5-point Likert scale. Lower satisfaction scores (1–3) were observed in patients who experienced flap loss or required prolonged follow-up for residual defects. The donor site generally bore a well-settled grafted scar, which was usually not conspicuous. (Figure 6) In some cases, secondary procedures such as flap thinning or web space widening were required. (Figure 7)

### Statistical analysis

• Peninsular (Type II and IV) and split-skin (Type Is and IIIs) flaps had better survival and fewer complications.
• Type IIIt flaps showed the highest complication rate, with an odds ratio (OR) of 5.0 (95 % CI: 0.3–7.7, *p* = 0.03), which was statistically significant compared to other flap types.
• Prolonged hospital stay was more frequently observed in Type IIIt flaps (OR = 5.0, *p* = 0.5), although this was not statistically significant.
• Due to the modest sample size, no statistically significant differences were observed in overall flap survival or hospital stay among the other flap types. (Tables 1, 2, and 3 provide further statistical details).

## Discussion

Reconstruction of soft tissue defects of the thumb is an arduous task due to the limited availability of local tissue. The preservation of length and sensibility are the fundamental objectives of thumb reconstruction. The first dorsal metacarpal artery (FDMA) flap, cross-finger flap, neurovascular island flap, and palmar advancement flap are some of the possible reconstructive options.^10^^,^^11^, ^12^ In addition, free tissue transfer from the first or second toe is another valid option, although it requires strong technical skill, increased logistical needs, sensory nerve coaptation, and success is never guaranteed.^13^^,^^14^

The first dorsal metacarpal artery, together with its corresponding veins and a branch of the superficial radial nerve, is harvested as part of the pedicled neurovascular flap from the dorsum of the index finger. It readily reaches the palmar or radial aspects as well as the thumb pulp because of its broad rotational arch. The accompanying veins of the FDMA provide excellent venous drainage. The size of the FDMA flap, along with its extended variety, is usually sufficient to cover various thumb defects, while donor site morbidity remains modest.

Numerous publications have discussed the application of the FDMA flap. Ratcliffe explained coverage of significant pulp deficiencies in the normal-length thumb using the FDMA flap.^15^ In a study of 23 patients, Sherif et al.^16^^,^^17^ reported the use of FDMA flaps for reconstruction of the first web space, the palmar and dorsal surface of the thumb, and even the dorsum of the hand in a bomb blast case. Eski et al.^18^ claimed excellent results in burn patients with thumb contractures treated with pedicled FDMA flaps.

It was El-Khatib who, in his research on thumb resurfacing, described the extended first dorsal metacarpal artery islanded flap.^19^ The author claimed that the abundant subdermal plexus supplying the dorsal skin makes this extension feasible. The extended first dorsal metacarpal artery islanded wraparound flap was also successfully used by Gebhart and Meissl for thumb reconstruction.^7^

Although FDMA flap surgeries have been conducted and reported in the literature in the past, we felt the need to consolidate these approaches into a simple, unified classification system. Our system incorporates distal extent, pedicle type (islanded/peninsular), and transfer method (tunneled/split skin). Importantly, subtypes It, Is, IIIt, and IIIs are not standalone classes but technical refinements within Types I and III, determined by flap tension, ease of inset, defect geometry, and surgeon experience. As reflected in our results, these choices significantly affect flap survival and complication rates. Table 4 compares this classification to prior known systems.

**Table 4:** Comparative table of FDMA flap Classification Systems.

System	Key features	Limitations	Advantages of our system
Foucher	Classic FDMA island flap up to PIP joint	Limited reach; higher congestion risk with tunneling	Integrates extended reach and simplifies selection
El-Khatib	Extended FDMA island flap beyond PIP, subdermal pedicle	Higher venous compromise; technically demanding	Adds peninsular option with skin bridge to improve safety
Yasir	Categorizes by extent, pedicle type, and transfer method	Newly proposed; requires broader validation	Simple, structured, guides flap choice, reduces complications

Our study showed that the traditional boundary of the PIP joint can be safely surpassed if certain modifications are undertaken to reduce complications. Extended FDMA flaps (Type III and IV) can cover almost any volar or dorsal thumb defect, including those near the tip. The results indicate that peninsular flaps had better survival rates and fewer complications compared to islanded flaps, and split-skin transfer had an advantage over tunneled transfer techniques. While Type IIIt flaps were associated with a significantly higher complication rate (*p* = 0.03), the corresponding confidence interval was wide, reflecting variability and limited sample size. The observed trend toward prolonged hospital stay in patients with Type IIIt flaps (OR = 5.0, *p* = 0.5) suggests a potential association that did not reach statistical significance, likely due to limited power.

These findings are supported by the study conducted by Jose Couceiro, who found that the classical racquet-shaped Holevich flap exhibited less flap necrosis and a lower incidence of venous congestion than the Foucher islanded flap.^20^ Similar results have been reported by Al Lahham, who demonstrated that designing a peninsular flap with a skin bridge and avoiding tunneling decreased the complication rate considerably and improved flap survival.^21^ Beyond the PIP joint, vascular variability increases, particularly in the subdermal and venous plexus, making islanded flaps more prone to venous congestion. Hence, the peninsular flap with a preserved skin bridge improves venous outflow and flap survival.

A proficient subdermal dissection while raising the islanded flap pedicle is of critical importance, as any damage to the underlying vein can result in flap failure. Peninsular flaps carry a lower risk of postoperative edema and congestion compared to islanded and tunneled flaps. Hence, Type III and Type IV flaps should be preferentially designed as peninsular when feasible to prevent complications.

Notably, surgeon experience and the learning curve for extended and tunneled flaps likely influenced outcomes in our series. These technical challenges should be explicitly considered when selecting the flap type, particularly for less experienced surgeons. Patient priorities were primarily centered on functional restoration rather than aesthetics, with most preferring defect coverage without thumb shortening. Only a minority expressed concern about the appearance of the reconstructed or donor site. Although formal functional outcome measures were not collected, follow-up observations indicated that patients generally regained functional pinch and grip strength sufficient for performing basic activities of daily living.

Strengths and Limitations of the Study: The primary strength of this study is the introduction of a novel classification system that consolidates various FDMA flap modifications into a clinically practical framework. This system offers a structured approach to flap selection, simplifying surgical decision-making. Additionally, the study compares different FDMA flap subtypes and provides insights into their outcomes and complications. However, the modest sample size (29 cases) and single-center design may limit the generalizability of the findings. A power analysis and multicenter collaboration would enhance the robustness of the conclusions. The study lacks follow-up data on objective functional outcomes, such as grip strength or two-point discrimination, and formal patient-reported outcome measures, such as the DASH score. Donor site morbidity (e.g., sensory changes or scar contracture) was not quantified and should be addressed in future research.

Future Directions: The proposed FDMA flap classification system lays a foundation for standardizing surgical planning for soft tissue reconstruction of the thumb. Future studies should aim to:

• Validate this classification system in larger, multicenter cohorts with diverse patient populations.
• Conduct long-term functional assessments, incorporating objective scoring systems for sensation, grip strength, and dexterity.
• Explore advanced imaging techniques (such as indocyanine green angiography) to optimize flap planning and improve outcomes.
• Assess the impact of surgeon experience and the learning curve on flap selection, complications, and success rates.
• Expand comparisons with prior classification systems (e.g., Foucher, El-Khatib) to highlight anatomical limitations and advantages.
• Integrate this classification into reconstructive hand surgery training to help young surgeons make informed, evidence-based decisions, ultimately leading to improved patient outcomes.

## Conclusion

The FDMA flap and its extended variations offer a versatile and reliable solution for thumb reconstruction. The proposed classification system provides a straightforward, clinically useful guide that helps surgeons select the appropriate flap based on defect requirements and their own expertise, leading to better functional and aesthetic outcomes. Our study demonstrates that peninsular flaps are more reliable especially when the flap must extend beyond the PIP joint to cover distal defects, using a peninsular flap with a skin pedicle improves survival and reduces complication rates

## Ethical approval

All necessary ethical clearances were obtained, and informed consent (including for photography) was taken from all participants. The STROBE guidelines were properly followed while formulating this research paper.

## Funding

No external funding was received for this study.

## Declaration of competing interest

The authors declare no conflict of interest.
